# Enhanced Absorption and Safety of MuscleBlaze CreAMP™: A Comparative Analysis With Regular Micronized Creatine Monohydrate in Healthy Male Adults

**DOI:** 10.7759/cureus.81555

**Published:** 2025-04-01

**Authors:** Anupam Trehan, Rachna Anand, Puja Kumari, Harinder Singh, Neha Soni, Ravij Madan, Raman Matta, Sameer Maheshwari, Manoj K Verma

**Affiliations:** 1 Strategy and Design, Bright Lifecare Pvt Ltd, Gurugram, IND; 2 Scientific Research, Bright Lifecare Pvt Ltd, Gurugram, IND; 3 Biotechnology Research, Bright Lifecare Pvt Ltd, Gurugram, IND; 4 Clinical Research, Bright Lifecare Pvt Ltd, Gurugram, IND; 5 Nutrition, Bright Lifecare Pvt Ltd, Gurugram, IND; 6 Ingredient Research, Bright Lifecare Pvt Ltd, Gurugram, IND; 7 Analytical Research, Bright Lifecare Pvt Ltd, Gurugram, IND; 8 Marketing Research and Management, Bright Lifecare Pvt Ltd, Gurugram, IND

**Keywords:** absorption, bioavailability, creamp™, muscle energy, performance, pharmacokinetics, safety, s: : creatine monohydrate, sports nutrition, supplementation

## Abstract

Background

Creatine monohydrate is a widely utilized dietary supplement in sports nutrition, valued for its role in enhancing muscle energy availability, power output, and performance during high-intensity, short-duration activities. Creatine monohydrate is effective but limited by absorption inefficiencies and side effects. Enhanced forms can improve uptake, reduce gastrointestinal discomfort, and optimize muscle energy utilization, meeting athletes’ evolving performance needs.

Methods

This study involved 32 healthy male volunteers aged 18-50 years, with a BMI of 18.5-25.0 kg/m² and body weight of ≥50 kg. This study evaluated the bioavailability and safety of MuscleBlaze Creatine Monohydrate (CreAMP™) (Bright Lifecare Pvt Ltd, Gurugram, India), containing 3.0 g creatine monohydrate and 0.1 g Creabsorb™ (Indian Patent: IN202311057466), against a standard 3.0 g micronized creatine dose. In a double-blind, randomized crossover trial (CTRI/2024/08/073021), 32 healthy males (18-50 years) received both formulations under fasting conditions. The study compared two oral creatine monohydrate formulations: CreAMP™ Micronized Creatine Monohydrate (test) and Regular Micronized Creatine Monohydrate (reference) (Bright Lifecare Pvt Ltd, Gurugram, India). Blood samples were collected pre-dose and up to six hours post-dose over two periods, separated by a washout period of one week. Pharmacokinetic parameters were analyzed using Phoenix® WinNonlin® 8.5 (Certara, Radnor, PA).

Results

CreAMP™ has significantly higher bioavailability, absorption, and plasma retention compared to the reference formulation. With a 38.97% increase in bioavailability, an 18.10% higher C_max_, a 21.37% longer half-life, 34.67% lower clearance, and a 10.13% higher mean residence time, CreAMP™ demonstrates superior pharmacokinetic properties. These findings suggest that CreAMP™ offers improved creatine uptake, sustained plasma levels, and the potential for reduced dosing frequency, making it a more effective formulation for creatine supplementation.

Conclusion

The study findings establish CreAMP™ as a superior creatine formulation, offering enhanced bioavailability, faster absorption, and prolonged plasma retention. These pharmacokinetic advantages indicate that CreAMP™ offers more efficient creatine uptake, improved energy availability, and optimized performance support for athletes.

## Introduction

Creatine monohydrate (CM) is one of the most extensively researched and widely used dietary supplements in sports nutrition. CM is known for its potential to enhance athletic performance and support muscle recovery; creatine plays a critical role in energy metabolism within the human body. Creatine functions primarily within the phosphagen energy system, which provides rapid energy for high-intensity, short-duration activities such as sprinting and weightlifting. Once ingested or synthesized endogenously, creatine is transported via the bloodstream to skeletal muscle, where approximately 95% of the body’s creatine is stored. Within the muscle, creatine is phosphorylated to form phosphocreatine, a high-energy compound. During intense physical activity, phosphocreatine donates its phosphate group to adenosine diphosphate (ADP) to rapidly regenerate adenosine triphosphate (ATP), the primary energy currency of the cell [1]. The human body stores creatine in both free and phosphorylated forms, with total creatine content in skeletal muscle ranging from 120 to 140 mmol/kg of dry muscle mass in individuals with normal dietary intake and without supplementation. These levels are influenced by factors such as age, sex, muscle mass, and dietary habits [2]. Creatine is synthesized endogenously in the liver, kidneys, and pancreas from the amino acids arginine, glycine, and methionine at a rate of approximately 1-2 g/day. An additional 1-2 g is typically obtained from dietary sources, primarily red meat and fish [3]. While the human body can endogenously synthesize creatine from amino acids, dietary sources, such as red meat and fish, often fail to provide sufficient amounts to meet the elevated demands of athletes. Consequently, supplementation with CM has become a widely adopted strategy for optimizing muscle energy stores and enhancing athletic performance. Supplementation with CM can significantly increase muscle creatine stores, with levels rising by 10-40% depending on the individual’s baseline creatine levels and dosage regimen [2]. A standard loading protocol involves consuming 20 g of CM per day, divided into four doses, for five to seven days, followed by a maintenance dose of 3-5 g/day. This strategy has been shown to elevate muscle phosphocreatine concentrations, enhancing the capacity for ATP resynthesis and improving performance in high-intensity, intermittent exercise tasks [4]. Furthermore, increased intramuscular creatine levels may provide additional benefits such as reduced muscle damage, improved recovery, and augmented adaptations to resistance training [5]. Studies have indicated that CM is not degraded during normal digestion and that nearly 99% of orally ingested CM is either taken up by muscle or excreted in urine. The upper limit of creatine storage appears to be about 160 mmol/kg of dry muscle mass in most individuals. About 1-2% of intramuscular creatine is degraded into creatinine (metabolic byproduct) and excreted in the urine [2]. Further, no medically significant side effects have been reported in the literature. Nevertheless, supplement manufacturers have continually introduced newer forms of creatine into the marketplace. These newer forms have been purported to have better physical and chemical properties, bioavailability, efficacy, and/or safety profiles than regular CM [6]. However, there is little to no evidence that any of the newer forms of creatine are more effective and/or safer than CM whether ingested alone and/or in combination with other nutrients [7]. One of the key limitations of CM is its bioavailability. While it is highly effective, only a fraction of ingested creatine is absorbed and utilized by the body due to capacity-limited absorption. CM, upon ingestion, undergoes passive diffusion and active transport processes to enter the bloodstream and, subsequently, the muscle cells. The creatine transporter (CreaT) protein mediates this uptake, but its capacity is finite, often resulting in a saturation effect where additional creatine intake offers diminishing returns. This limited absorption contributes to inefficiencies, requiring athletes to consume higher doses to achieve optimal muscle creatine levels [8,9]. Another factor affecting bioavailability is the physicochemical properties of CM. It has relatively low solubility in water, which can hinder its complete dissolution and absorption in the gastrointestinal tract. As a result, some of the ingested creatine remains unabsorbed and may cause gastrointestinal discomfort, including bloating, cramping, or diarrhea. This is particularly common during the "loading phase," where athletes consume large doses (typically 20-25 g/day) over five to seven successive days to saturate their muscle creatine stores [10].

The standard creatine loading protocol, while effective, poses challenges for athletes due to its demanding dosing schedule. A standard regimen involves a loading phase of high doses (20-25 g/day) spread across multiple servings per day, followed by a maintenance phase of 3-5 g/day [10]. While this approach accelerates muscle saturation, the frequent and high-dose intake required during the loading phase can be inconvenient and may lead to gastrointestinal side effects, especially for individuals with sensitive digestive systems [11].

Additionally, the rapid clearance of creatine from the bloodstream further necessitates frequent dosing. Creatine has a relatively short plasma half-life, meaning it is quickly removed from circulation and stored in muscle tissue or excreted as creatinine. This characteristic requires consistent supplementation to maintain elevated plasma levels, adding to the complexity and inconvenience of standard dosing protocols. For athletes juggling intense training schedules, this frequent loading can become a logistical and practical burden [12].

Emerging solutions for enhanced bioavailability

Given the limitations associated with standard supplementation, researchers and manufacturers have explored innovative formulations to improve their absorption and overall efficacy. Enhanced creatine formulations aim to address issues related to solubility, uptake, and retention, thereby providing athletes with a more efficient and convenient supplementation strategy. Novel forms, such as creatine hydrochloride (HCl), creatine ethyl ester (CEE), and buffered creatine, address issues with solubility and reduce gastrointestinal discomfort associated with standard CM [13]. These formulations enhance absorption and muscle saturation by increasing the content of creatine available for absorption while minimizing side effects [13]. However, they may come with limitations, including higher costs, limited long-term research on their efficacy, and the potential for lower overall effectiveness compared to CM, which remains the gold standard for creatine supplementation. To overcome the challenges of the gold standard, one such formulation has been formulated, i.e., MuscleBlaze Creatine Monohydrate (CreAMP™), which combines 3.0 g of CM with 0.1 g of Creabsorb™ (Indian Patent Application: IN202311057466). Creabsorb™ is an absorption-enhancing compound designed to improve the solubility and efficacy of CM. By facilitating more efficient uptake into the bloodstream and muscle cells, CreAMP™ offers several potential advantages over Regular Micronized Creatine Monohydrate formulations, such as increased endurance and athletic performance due to its enhanced uptake.

Study objectives and rationale

The primary objective of this study was to evaluate the pharmacokinetics and safety of CreAMP™ compared to an RMCM formulation. Key pharmacokinetic parameters, including maximum plasma concentration (C_max_) and area under the curve (AUC₀-∞). Secondary pharmacokinetic parameters, including elimination half-life (t½) and clearance, were assessed to determine the extent of absorption and retention of creatine in the body.

CreAMP™ may enhance tolerability, making creatine supplementation more accessible to a broader range of individuals, including those with a sensitive gut. Enhanced creatine formulations, such as CreAMP™, offer several potential advantages for athletes and fitness enthusiasts. The formulations improve the solubility and uptake of creatine, maximizing the amount available for muscle storage and minimizing wastage. They also provide the benefit of reduced dosing frequency due to prolonged plasma retention and a longer elimination half-life, which eliminates the need for a rigorous loading phase and simplifies supplementation routines. Additionally, lower total doses and improved gastrointestinal compatibility enhance tolerability, reducing the risk of side effects and making creatine supplementation more comfortable and convenient. Finally, the improved absorption and retention of these formulations optimize muscle creatine saturation, allowing athletes to experience performance benefits more quickly and efficiently [13].

## Materials and methods

MuscleBlaze CreAMP™ and RMCM were manufactured and packed by Bright Lifecare Pvt Ltd in Gurugram, India. The material was supplied in premeasured individual dose packets of 3.1 g and 3 g, respectively, also by Bright Lifecare Pvt Ltd.

Experimental design

The study was planned as a double-blind, randomized, two-arm, two-period, crossover, oral bioavailability study. The study was conducted in healthy, adult, human male subjects under fasting conditions. As per the study protocol approved by the Independent Ethics Committee (IEC) (ECR/1581/Inst/RJ/2021), 32 subjects were enrolled in the study.

All subjects were divided into two equal groups comprising 16 subjects each. The two groups were assigned to either treatment A or treatment B and sequenced (AB and BA) in a random order according to the randomization schedule generated using statistical techniques with SAS® statistical software (version 9.4; SAS Institute Inc., USA). Treatment A consisted of administering 3.1 g of MuscleBlaze CreAMP™, while treatment B consisted of administering 3 g of RMCM. The difference in the rate of creatine absorption was determined by quantifying the serum levels of creatine.

Subjects

A total of 32 volunteers were selected randomly from the volunteer database. The volunteer database was managed using the Electronic Clinical Data Management System (eCDMS), which facilitates secure storage of participant records, eligibility verification, and study progress tracking. It also underwent a standardized screening procedure. Thirty-two healthy, lean males aged 18-50 years, each with a body mass index (BMI) ranging from 18.5 to 25, volunteered for this study. None of the subjects took any specific creatine-rich dietary regimen, muscle-toning, or bodybuilding program during the study. The clinical study was enrolled in the Clinical Trials Registry - India (CTRI/2024/08/073021). Each subject was informed of the purpose, methods, and probable risks associated with the study, and informed consent was signed by each participant.

Dietary protocol

An overnight fast of at least 12 hours was mandatory before every treatment. All participants were advised to resume their diet after the last blood sample and strictly follow the prescribed diet and dietary restrictions as per the approved study protocol.

Study protocol

Subjects received treatment in a crossover design under fasting conditions as per the randomization schedule. All subjects received 3.1 g of MB CreAMP™ or 3 g of RMCM with 240 mL of drinking water at room temperature in a sitting position and under the supervision of trained study personnel after an overnight fasting of at least 12 hours in each period. The time of dosing was recorded. The dosing was performed under the supervision of the investigator, authorized study personnel, and quality assurance (QA) personnel. Subjects were not allowed to drink water for one hour before dosing until two hours post-dose. Food was prohibited until six hours after dosing. Subjects remained in the seated or semi-inclined position until two hours after dosing. The two treatment days were separated by a washout period of seven days. The administration of the investigated product was performed as per the randomization schedule generated using SAS® statistical software (version 9.4 or higher; SAS Institute Inc., USA) and as authorized by a biostatistician.

A total of eight venous blood samples were collected at the following times: 00.00 (pre-dose), 00.50, 01.00, 01.50, 02.00, 03.00, 04.00, and 06.00 hours post-dose. Samples were taken via an indwelling cannula placed in the forearm vein/dorsal aspect of the hand. Pre-dose blood samples were collected within two hours prior to product administration, with a window period of +02 minutes for all post-dose samples. The pre-dose blood sample was 5 mL, and post-dose samples were 6 mL each, collected using gel clot activator vacutainers. These blood samples were then analyzed to measure creatine levels.

Sample collection and preparation

Blood samples were collected in pre-labeled gel clot activator vacutainers. The vacutainers were gently inverted up-down manually five times after collection to mix the tube clot activator with blood and placed at room temperature. Then, allow the blood to clot by leaving it undisturbed at room temperature for 30 minutes until centrifugation. Blood samples are placed in a refrigerated centrifuge and then spun at a set temperature of 2°C to 10°C at 4,000 ± 100 rpm for 10 minutes. The serum separated and removed the clot. After centrifugation, the separated serum was transferred to suitably pre-labeled polypropylene tubes in duplicate (approximately 1 mL serum in primary aliquot and remaining volume in secondary aliquot), placed in a wet ice bath and stored upright in a box containing dry ice or in a deep freezer at -70 °C ± 10°C for interim storage. Finally, the samples were transferred to an analytical laboratory and stored in a deep freezer at -70°C ± 10°C until completion of analysis.

Analytical method

The blinded samples of serum were submitted to the laboratory. Creatine analysis consisted of the quantification of serum creatine levels for each patient at each time point. The analysis was performed using a standard creatine microplate assay kit from Biorbyt (Cambridge, UK). Quantification was performed by comparing it with the reference standard of the kit. Creatine levels are reported in nanomoles per liter as the percentage increases in the area under the concentration-time curve (AUC).

Statistical analysis

All significance and power testing on the results were performed at an alpha level of 0.05. The within-group analysis was performed between the two treatment groups. The mean AUC and other pharmacokinetic parameters were calculated using Phoenix® WinNonlin® version 8.5 (Certara, Radnor, PA). The results are expressed as the mean ± SD. The p-values were calculated using t-test.

## Results

The study confirmed reliable pharmacokinetic comparisons between MB CreAMP™ and RMCM. Conducted with strict methodological standards and adherence to protocols, the results suggest that both substances are safe and effective supplements for healthy individuals.

**Figure 1:**
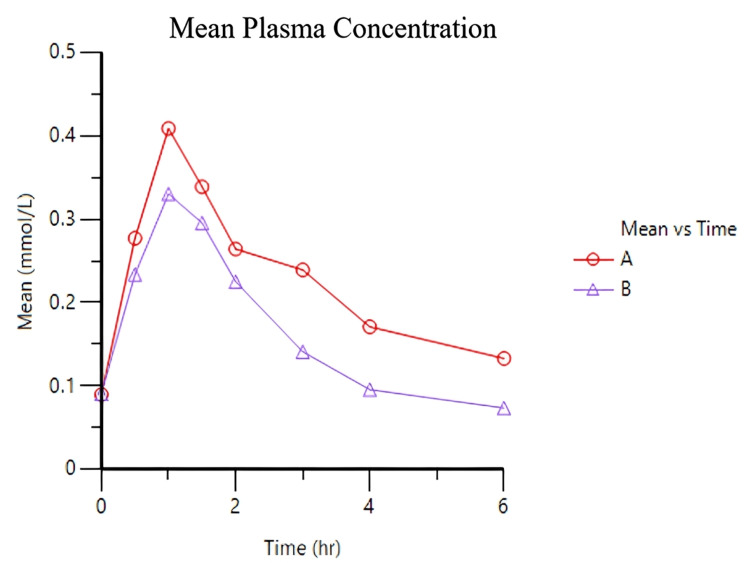
Mean plasma concentration-time curve showing absorption and elimination phases CreAMP™ (sample A-line plot with circular data points ): Mean plasma drug concentration-time curve for CreAMP™, illustrating absorption and elimination phases with peak C_max_. RMCM (sample B-line plot with triangle data points): Mean plasma drug concentration-time curve for RMCM, showing its corresponding C_max_. C_max_: Maximum plasma concentration point for each formulation, indicating the extent of absorption. T_max_: Time to reach C_max_, reflecting the rate of absorption. Statistical significance: t-test was used to calculate statistical significance. The result (p-value < 0.001) indicates a significant difference between CreAMP™ and RMCM. X-axis: Time post-administration (hours). Y-axis: Plasma creatine concentration (mmol/L).

**Figure 2:**
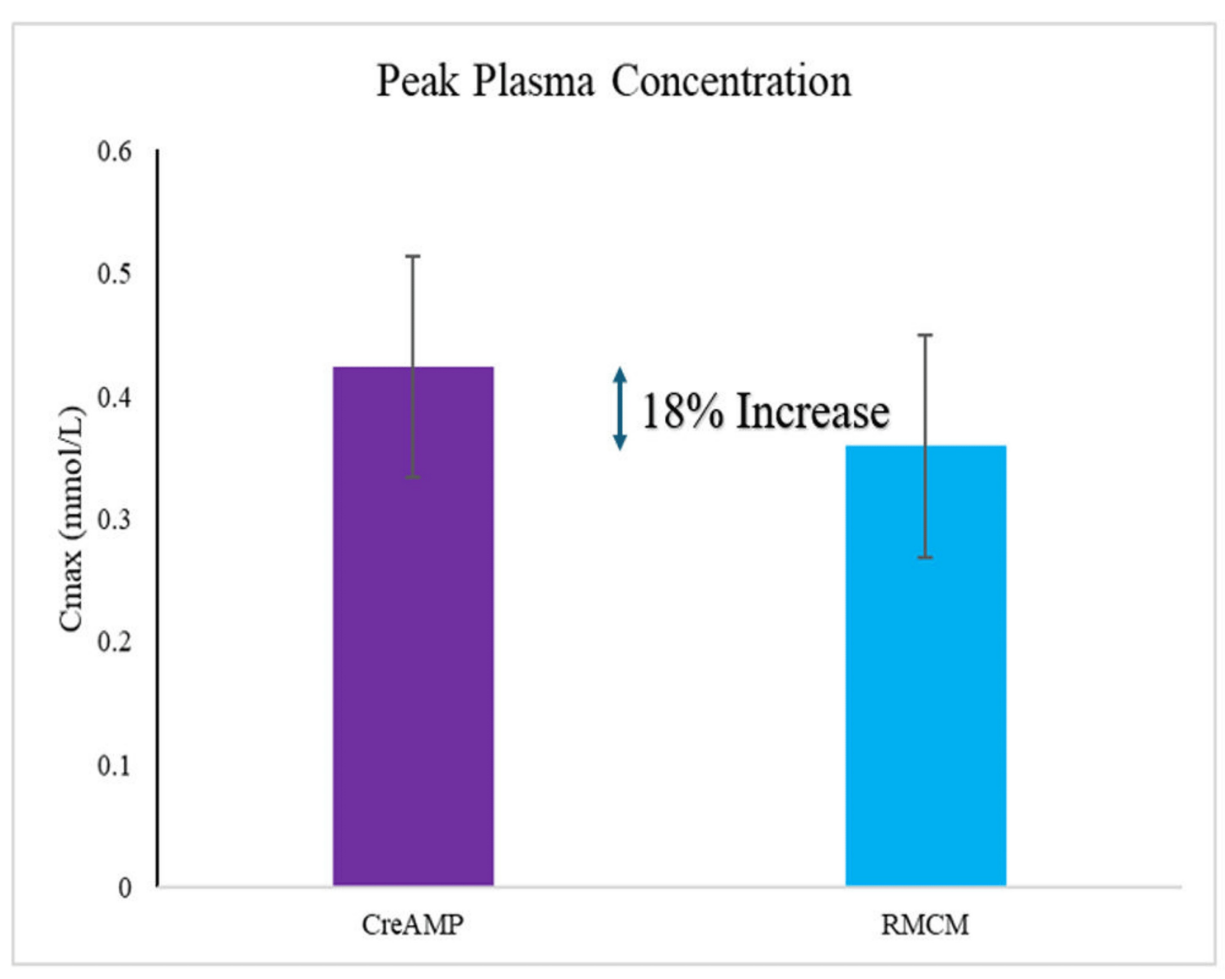
Peak plasma concentration (Cmax) bar graph CreAMP™: Represents the mean plasma concentration-time profile of CreAMP™, highlighting its C_max_ (maximum plasma concentration) for comparison. RMCM: Depicts the mean plasma concentration-time profile of RMCM, indicating its C_max_. C_max_: The peak plasma concentration observed for each formulation, reflecting the rate and extent of absorption. Statistical significance: t-test was used to calculate statistical significance. The result (p-value 0.01) indicates a significant difference between CreAMP™ and RMCM. Plasma concentration (Y-axis): Measured creatine levels in plasma in (mmol/L).

**Figure 3:**
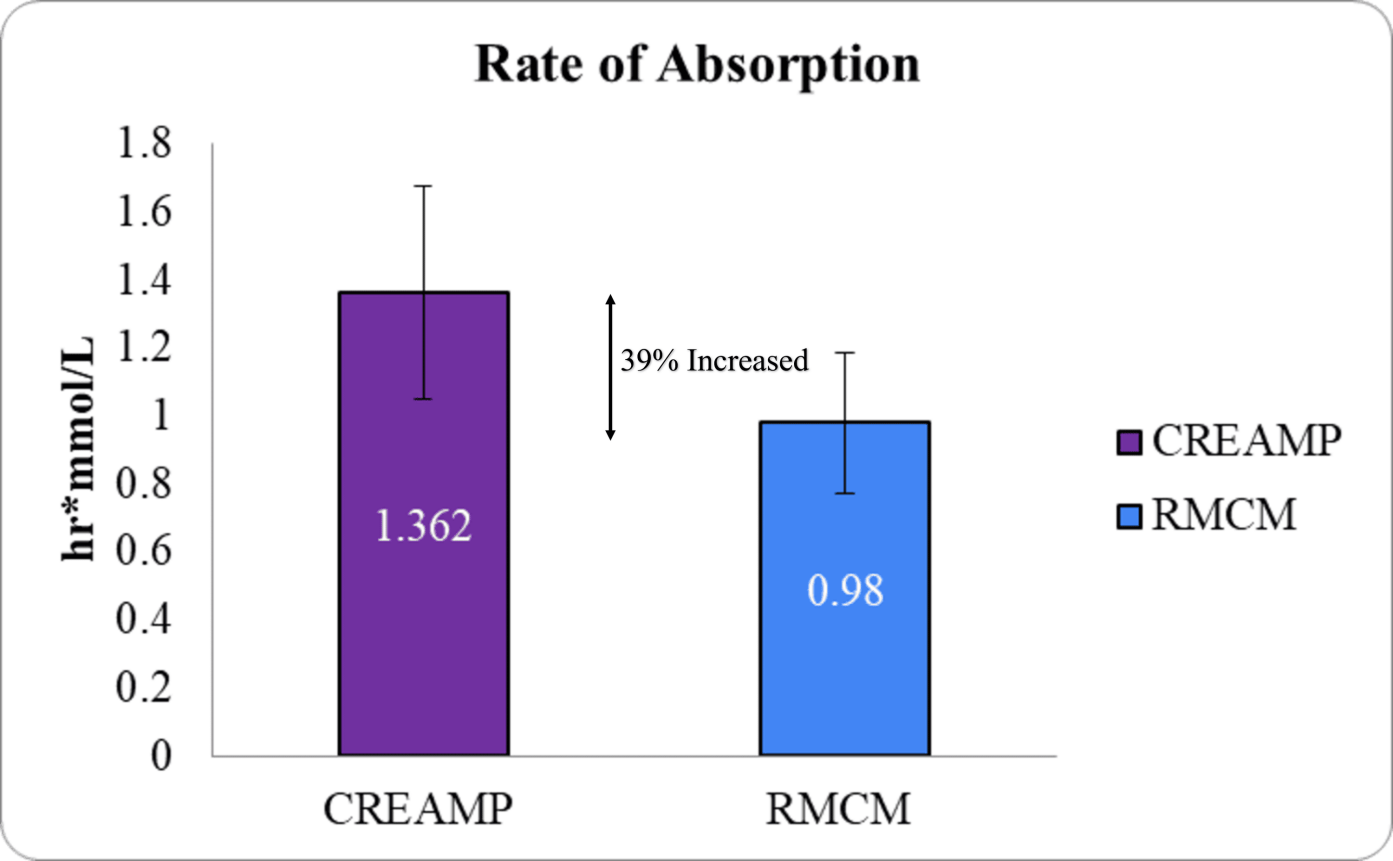
Rate of absorption (AUC0-t ) bar graph (p-value <0.001) CreAMP™: Represents the rate of absorption for CreAMP™, measured by the area under the curve from time zero to the last measurable concentration (AUC_₀-t_). RMCM: Indicates the rate of absorption for RMCM based on its AUC_₀-t_. AUC_₀-t_ (area under the curve): Reflects the total drug exposure over time, representing the extent of absorption. Statistical significance: t-test was used to calculate statistical significance. The result (p-value < 0.001) indicates a significant difference between CreAMP™ and RMCM. X-axis: Test formulations (CreAMP™ vs. RMCM). Y-axis: AUC_₀-t_ values (hr.mmol/L), representing the extent of absorption.

Notably, the findings revealed that CreAMP™ demonstrated an 18.10% greater peak plasma concentration (Figure 1 and Figure 2) and a 38.97% improvement in bioavailability when compared to RMCM (Figure 3).

Both products effectively maintained plasma creatine levels within normal physiological ranges across all time points. This includes pre-dose and six hours post-dose. This consistency highlights its potential as a more efficient creatine supplement option. We want to emphasize that both CreAMP™ and RMCM were well-tolerated. We observed no significant adverse effects, suggesting a strong safety profile for both formulations.

**Figure 4:**
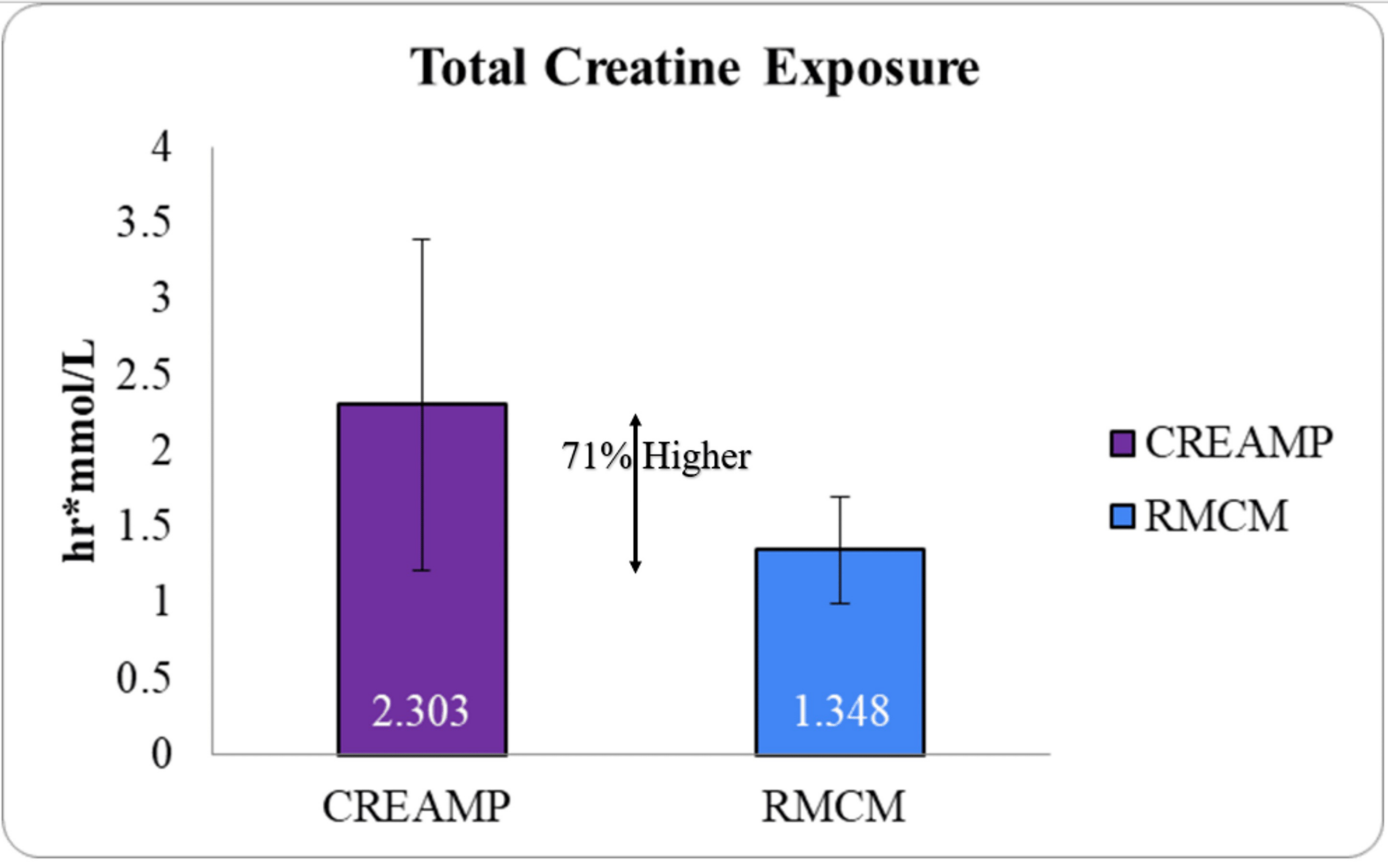
Total creatine exposure (AUC 0-inf) (p-value < 0.001) CreAMP™: Represents the total creatine exposure for CreAMP™, measured by the area under the curve from time zero to infinity (area under the curve (AUC)_₀-∞_), indicating overall creatine absorption. RMCM: Shows the total creatine exposure for RMCM, based on its AUC_₀-∞_. AUC_₀-∞_: Reflects the total drug exposure over time, representing both the extent and duration of absorption until complete elimination. Line bar: Indicate the absorption difference, highlighting CreAMPTM having more total creatine exposure across samples. Statistical significance: t-test was used to calculate statistical significance. The result (p-value < 0.001) indicates a significant difference between CreAMP™ and RMCM. X-axis: Test formulations (CreAMP™ vs. RMCM). Y-axis: AUC_₀-∞_ values (hr.mmol/L), representing total creatine exposure.

Focusing on pharmacokinetics, CreAMP™ showed a 71% increase in total creatine exposure (AUC_₀-∞_) when compared to the reference product (Figure 4). This increase suggests that CreAMP™ ensures a more efficient supply of creatine, which is crucial for supporting muscle energy, strength, and recovery.

**Figure 5:**
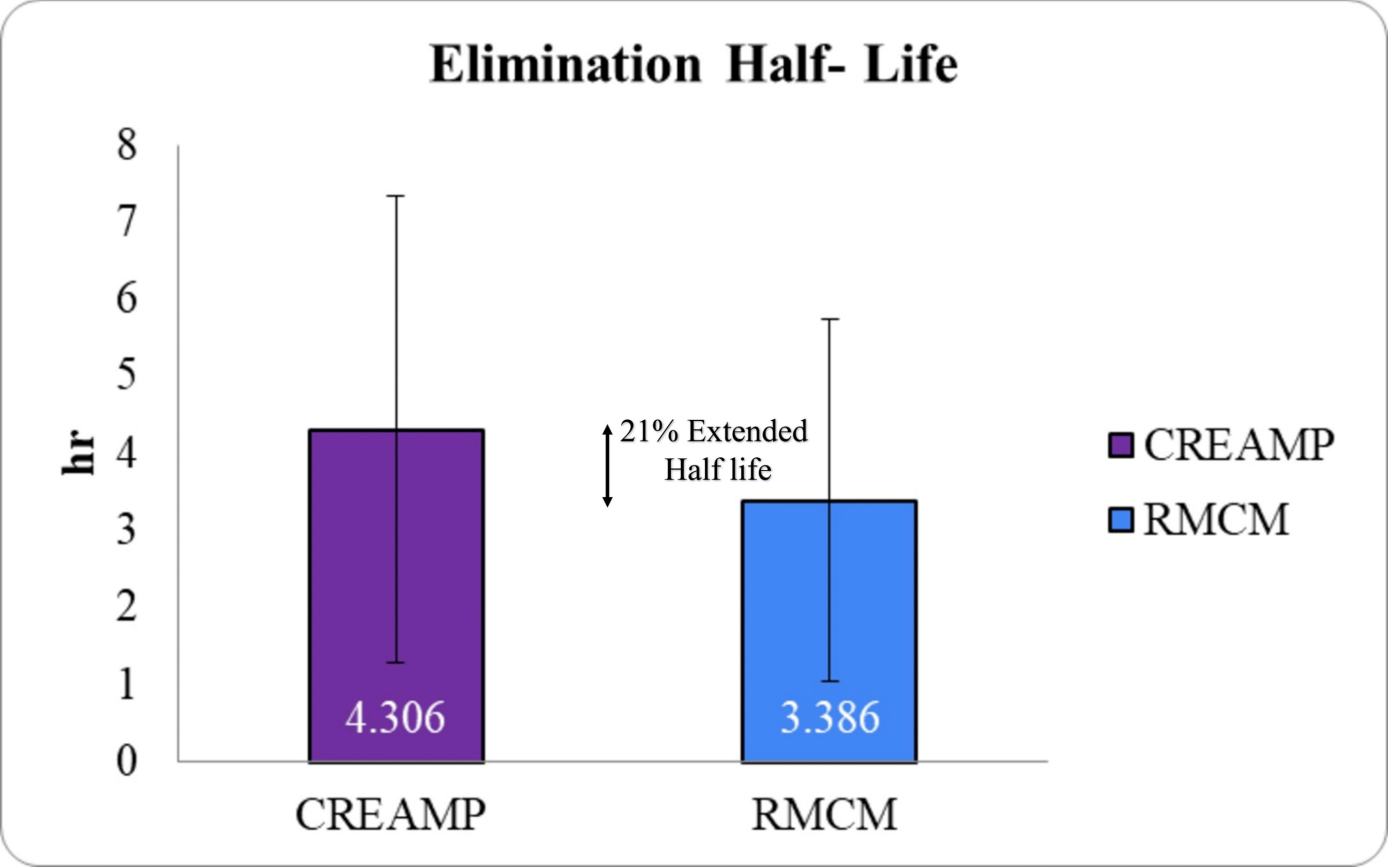
Comparison of elimination half-life (T½) between CreAMP™ and RMCM (p-value 0.0983) CreAMP™: Represents the elimination half-life (T½) of CreAMP™, indicating the time required for the plasma creatine concentration to decrease by 50%. RMCM: Shows the elimination half-life (T½) of RMCM for comparison. T½ (elimination half-life): Reflects the rate at which creatine is eliminated from the body, measured in hours. Statistical significance: t-test was used to calculate statistical significance. The result (p-value 0.0983) indicates statistically no significant difference between CreAMP™ and RMCM. X-axis: Test formulations (CreAMP™ vs. RMCM). Y-axis: Elimination half-life (T½) in hours.

**Figure 6:**
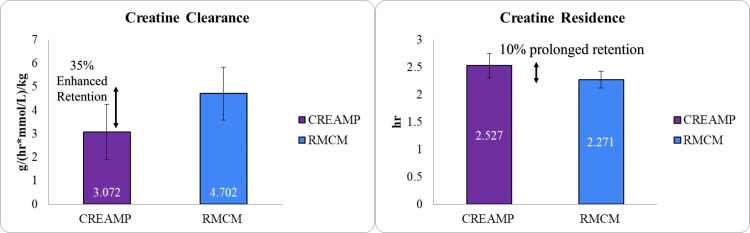
Comparison of clearance rate (CL) and mean residence time (MRT) between CreAMP™ and RMCM (p-value 0.0000) Statistical significance: t-test was used to calculate statistical significance. The result (p-value 0.0000) indicates a significant difference between CreAMP™ and RMCM. X-axis: Test formulations (CreAMP™ vs. RMCM) grouped by CL and MRT. Y-axis: for CL, clearance rate g/(hr*mmol/L)/kg; for MRT, mean residence time (hours)

**Figure 7:**
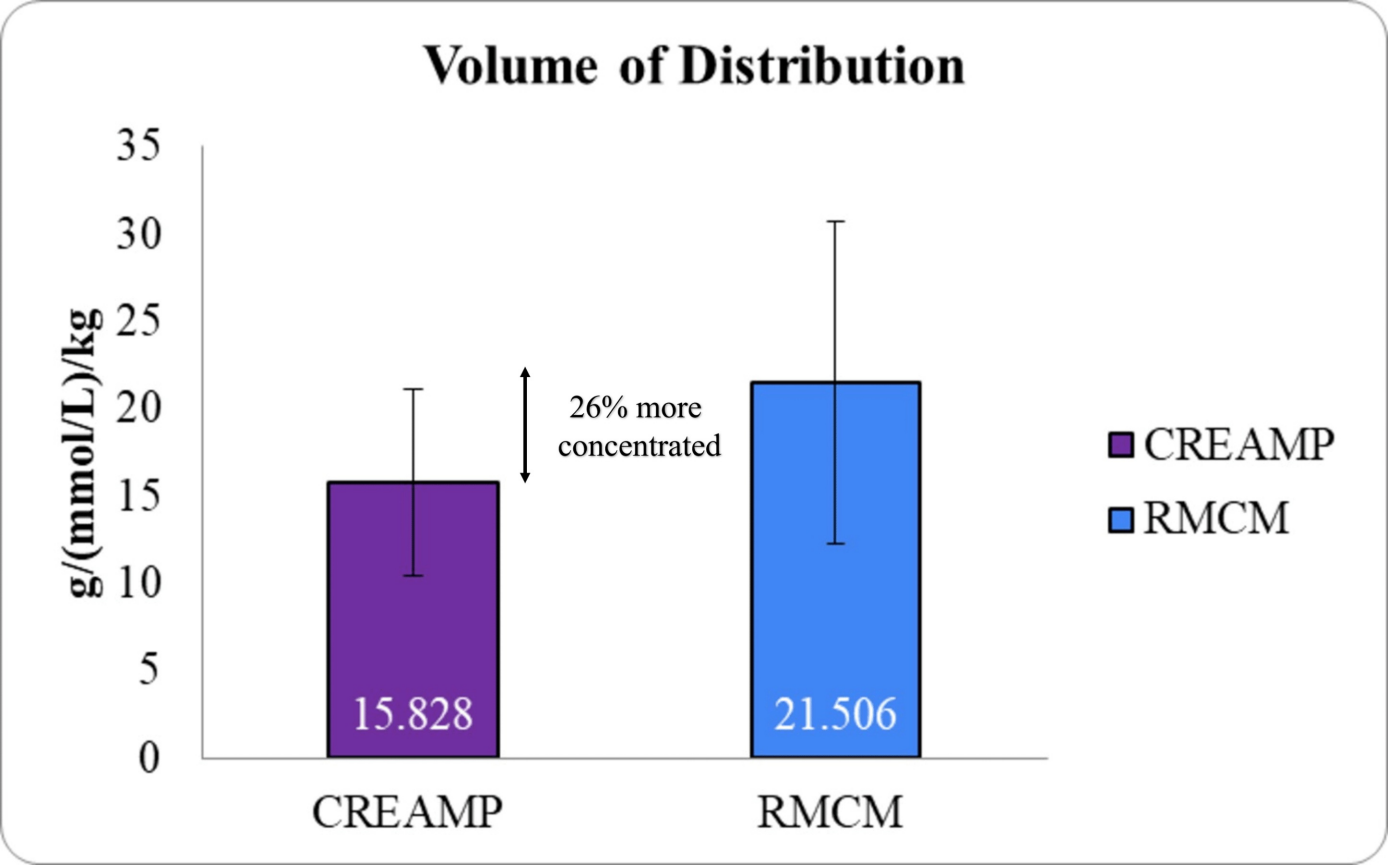
Comparison of volume of distribution (Vz) between CreAMP™ and RMCM (p-value 0.0001) CreAMP™: Represents the volume of distribution (Vz) for CreAMP™, indicating the extent to which creatine is distributed throughout body tissues. RMCM: Shows the Vz for RMCM for comparison. Vz (volume of distribution): Reflects the hypothetical volume in which creatine would need to be uniformly distributed to produce the observed plasma concentration, measured in g/(mmol/L)/kg. Statistical significance: t-test was used to calculate statistical significance. The result (p-value 0.0001) indicates a significant difference between CreAMP™ and RMCM. X-axis: Test formulations (CreAMP™ vs. RMCM). Y-axis: Volume of distribution (Vz) in g/(mmol/L)/kg. Additionally, CreAMP™ showed a 15.81% lower elimination rate constant (Kel) (Figure 8) and a 10.13% higher mean residence time (MRT) (Figure 6), prolonging its duration of action and enhancing creatine accumulation efficiency.

The half-life of CreAMP™ was 4.31 hours, which is 21.37% longer than the 3.39 hours observed with RMCM (Figure 5), allowing for sustained plasma creatine levels and reducing the frequency of dosing required during the loading phase. CreAMP™ also demonstrated a 34.67% lower clearance rate (Figure 6) and a 26.40% reduced volume of distribution (Figure 7), indicating less elimination and greater systemic retention of creatine for muscle uptake.

**Figure 8:**
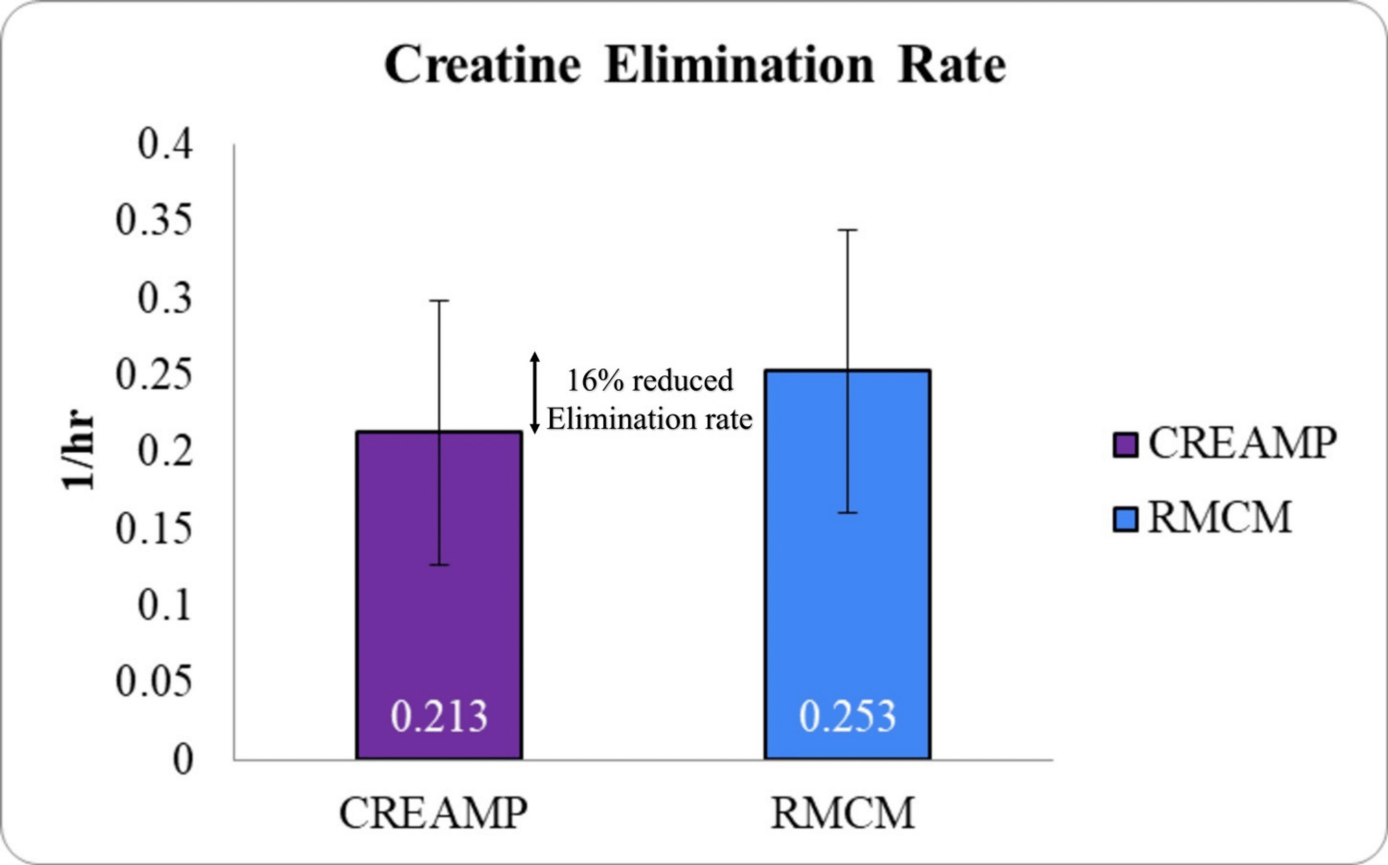
Comparison of elimination rate constant (Kel) between CreAMP™ and RMCM (p-value 0.0461) CreAMP™: Represents the elimination rate constant (Kel) for CreAMP™, indicating the rate at which creatine is eliminated from the body. RMCM: Shows the Kel for regularized micronized creatine monohydrate for comparison. Kel (elimination rate constant): Reflects the rate of drug elimination per unit of time, measured in hr^-^¹ (per hour). Statistical significance: t-test was used to calculate statistical significance. The result (p-value 0.046) indicates a significant difference between CreAMP™ and RMCM. X-axis: Test formulations (CreAMP™ vs. RMCM). Y-axis: Elimination rate constant (Kel) in hr^-^¹

Additionally, CreAMP™ showed a 15.81% lower elimination rate constant (Kel) (Figure 8) and a 10.13% higher mean residence time (MRT) (Figure 6), prolonging its duration of action and enhancing creatine accumulation efficiency.

The study indicates that CreAMP™ has better pharmacokinetic properties than RMCM. Due to its enhanced bioavailability, sustained plasma levels, and improved systemic availability, CreAMP™ appears to be a promising alternative for efficient creatine supplementation, especially in terms of dosing convenience and creatine loading potential.

## Discussion

The present study demonstrated that MuscleBlaze CreAMP™ exhibits superior pharmacokinetic properties compared to conventional CM. Notably, CreAMP™ achieved an 18.10% higher peak plasma concentration (C_max_) and a 38.97% increase in bioavailability, resulting in a 71% enhancement in total creatine exposure (AUC_₀-∞_). These findings suggest that CreAMP™ facilitates more efficient systemic delivery and retention of creatine, which may translate into improved muscle saturation and performance benefits over time. In addition to enhanced absorption, CreAMP™ displayed a 21.37% longer half-life and a 34.67% lower clearance rate, ensuring sustained plasma creatine levels. The observed 26.40% reduction in volume of distribution and 15.81% lower elimination rate further indicate improved retention and utilization. These pharmacokinetic advantages may allow for prolonged availability of creatine, potentially reducing the frequency and dosage required to maintain optimal levels.

Previous studies on CM have consistently shown that it reaches peak plasma concentration within one to two hours post-ingestion. Research by Buford et al. and Persky et al. confirmed CM’s rapid absorption and near-complete bioavailability [2,8], while Jäger et al. showed no significant physiological advantage of other creatine formulations over CM [7]. Jagim et al., in their study comparing buffered creatine to CM, showed no significant differences in muscle creatine content, body composition, strength, or anaerobic capacity, suggesting no advantage of buffered creatine over CM [14]. Antonio et al. further validated creatine’s effectiveness, showing significant improvements in muscle strength, hypertrophy, and athletic performance, even in untrained individuals [15]. This reinforces CM’s established role as a reliable performance-enhancing supplement.

**Figure 9:**
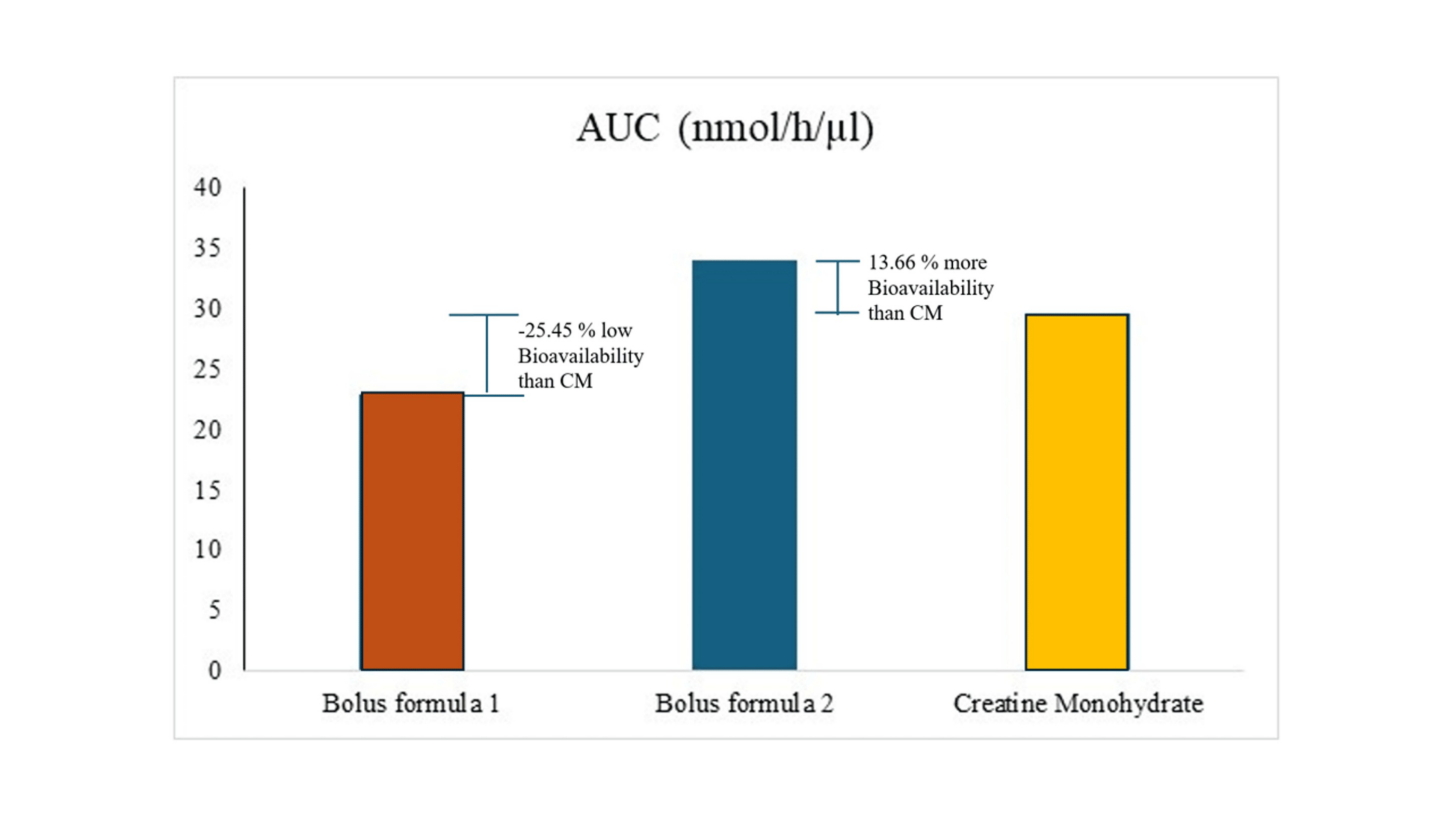
Area under the curve (AUC) (bioavailability) of creatine monohydrate and other bolus formula Bolus formula 1 (CreaBev 1) and bolus formula 2 (CreaBev 2) refer to formulations described in the study by Antonio et al. (2022) [15]. CreaBev 1 and CreaBev 2 are investigational creatine-containing formulations evaluated for their pharmacokinetic properties.

In addition, Antonio et al. evaluated the pharmacokinetics of two different 5 g bolus creatine formulations against 5 g CM, as represented in Figure 9. Their findings revealed that one formulation absorbed 25.45% less, while the other demonstrated 13.6% better absorption compared to regular CM [15]. CreAMP™ demonstrates superior bioavailability with higher C_max_, extended T_max_, and greater AUC, indicating enhanced absorption by 38.97% compared to RMCM. While CreaBev 1 shows moderate bioavailability with faster clearance, CreaBev 2, as described by Antonio et al. [15], improves upon this with better systemic exposure but still falls short of CreAMP™.

These results align with the notion that while creatine supplementation remains effective, the formulation can influence its bioavailability and absorption rates. Optimizing nutrient absorption is critical for maximizing efficacy, whether for creatine or protein supplementation. While Antonio et al. [15] examined differences in absorption rates between creatine formulations, the present study highlights how CreAMP™ enhances pharmacokinetic parameters, leading to more efficient creatine loading, absorption, and retention. This translates to superior benefits in dosing convenience, sustained plasma levels, and performance enhancement compared to regular CM and other creatine formulations. A similar approach has been applied in protein supplementation, as seen in the study on whey protein bioavailability enhancer - MB EnzymePro® (Bright Lifecare Pvt Ltd). By using a proprietary enzyme blend, MB EnzymePro® improved whey protein absorption by 50%, leading to better bioavailability of branched-chain amino acids (BCAAs) and enhanced nitrogen balance [16]. Just as CreAMP™ ensures better creatine delivery and systemic availability and MB EnzymePro® optimizes protein utilization, both formulations showcase the importance of scientifically engineered formulations that can significantly outperform generic alternatives. These findings underscore the importance of well-researched, clinically tested formulations in enhancing nutrient absorption and overall effectiveness. However, one limitation of the present study was the execution of the protocol under fasting conditions, whereas some studies have incorporated meals during dosing intervals.

Further research with a larger and more diverse population, along with the effect of meals, would help confirm the extended findings of the present study. Future studies can also include comparisons of CreAMP™ with several marketed forms of creatine supplementation and evaluate their effect on specific performance parameters among athletic populations.

## Conclusions

The findings of this study demonstrate that CreAMP™ exhibits superior pharmacokinetic properties compared to RMCM, achieving significantly higher bioavailability and peak plasma concentration. These factors contribute to enhanced systemic absorption and retention, potentially leading to greater muscle saturation over time. The formulation’s extended half-life and reduced clearance rate further support sustained plasma creatine levels, enhancing its overall effectiveness as a dietary supplement. Additionally, CreAMP™ presents a favorable safety profile, reinforcing its potential as a reliable and well-tolerated alternative to conventional creatine supplements. Its combination of superior absorption, prolonged systemic availability, and improved retention makes it an appealing option for individuals seeking to maximize creatine’s ergogenic benefits with reduced dosing frequency.

While these findings highlight the advantages of CreAMP™, further research is necessary to fully understand its long-term effects on muscle function, athletic performance, and overall muscle health. Future studies should assess its efficacy across diverse populations, including trained and untrained individuals, and explore its potential applications in clinical settings, such as muscle-wasting conditions or age-related sarcopenia. Comparative studies evaluating CreAMP™ against other emerging creatine formulations would also provide valuable insights into its real-world applicability. With further validation through long-term research, CreAMP™ has the potential to become a leading creatine supplement, benefiting both recreational and professional athletes, as well as individuals aiming to optimize muscle performance and recovery.
